# Mechanisms Underlying Interferon-γ-Induced Priming of Microglial Reactive Oxygen Species Production

**DOI:** 10.1371/journal.pone.0162497

**Published:** 2016-09-06

**Authors:** Nicholas G. Spencer, Tom Schilling, Francesc Miralles, Claudia Eder

**Affiliations:** 1 Infection and Immunity Research Institute, St. George’s University of London, London, United Kingdom; 2 Molecular and Clinical Sciences Research Institute, St. George’s University of London, London, United Kingdom; 3 Institute for Medical and Biomedical Education, St. George’s University of London, London, United Kingdom; Uniformed Services University, UNITED STATES

## Abstract

Microglial priming and enhanced reactivity to secondary insults cause substantial neuronal damage and are hallmarks of brain aging, traumatic brain injury and neurodegenerative diseases. It is, thus, of particular interest to identify mechanisms involved in microglial priming. Here, we demonstrate that priming of microglia with interferon-γ (IFN γ) substantially enhanced production of reactive oxygen species (ROS) following stimulation of microglia with ATP. Priming of microglial ROS production was substantially reduced by inhibition of p38 MAPK activity with SB203580, by increases in intracellular glutathione levels with N-Acetyl-L-cysteine, by blockade of NADPH oxidase subunit NOX2 activity with gp91ds-tat or by inhibition of nitric oxide production with L-NAME. Together, our data indicate that priming of microglial ROS production involves reduction of intracellular glutathione levels, upregulation of NADPH oxidase subunit NOX2 and increases in nitric oxide production, and suggest that these simultaneously occurring processes result in enhanced production of neurotoxic peroxynitrite. Furthermore, IFNγ-induced priming of microglial ROS production was reduced upon blockade of Kir2.1 inward rectifier K^+^ channels with ML133. Inhibitory effects of ML133 on microglial priming were mediated via regulation of intracellular glutathione levels and nitric oxide production. These data suggest that microglial Kir2.1 channels may represent novel therapeutic targets to inhibit excessive ROS production by primed microglia in brain pathology.

## Introduction

Production of large amounts of reactive oxygen species (ROS) and subsequent oxidative stress play a pivotal role in neurological diseases, while activated microglial cells are the major source of ROS production in brain pathology [1, 2]. Although ROS can have beneficial roles via regulation of cellular signaling mechanisms [3], excessive ROS production by microglia has detrimental effects on surrounding neurons via oxidative damage of neuronal lipids, proteins and DNA. Furthermore, intracellularly produced ROS can contribute to microglial neurotoxicity by enhancing the production of proinflammatory substances [2, 4, 5].

Activation of microglia with an appropriate stimulus, such as ATP [6], induces NADPH oxidase activity leading to the production of a certain amount of ROS. Pre-exposure of microglia to various agents, which do not cause ROS production themselves, can lead to significant enhancement of ROS production upon subsequent stimulation, e.g., with ATP. This process is known as "priming" [7]. Agents capable of priming microglial ROS production include amyloid-β [8–10], cytokines, such as interferon-γ (IFNγ) [11, 12] and tumor necrosis factor-α [11, 12], HIV-1 Nef protein [13], paraquat [14] and others. Priming agents, which do not induce, but potentiate ROS production have first been identified and subsequently thoroughly investigated in neutrophils [7, 15]. The number of substances causing priming of ROS production by neutrophils [7] is larger than that of priming agents found to date in microglia. However, it can be expected that the list of microglial priming agents will further expand due to the growing interest in microglial priming and activation.

Microglial priming represents one of the mechanisms leading to excessive ROS production and subsequent neuronal damage in brain pathology [1]. It is now well recognized that brain aging, traumatic brain injury and neurodegenerative diseases lead to the formation of primed microglia [1, 16, 17], while the proinflammatory cytokine IFNγ has been identified as a microglial priming factor. Under pathological conditions, infiltration of IFNγ-producing T cells in the brain is enhanced due to brain damage or aging-associated increased permeability of the blood brain barrier. Consequently, enhanced IFNγ concentrations have been found in the aged brain [18], following traumatic brain injury [19] and at early stages of neurodegenerative diseases, including Alzheimer’s disease [20], Parkinson’s disease [21] and vascular dementia [22].

In this study, we investigated IFNγ-induced priming of microglial ROS production. We identified mechanisms underlying this priming process and suggest that microglial Kir2.1 channels represent potential therapeutic targets to reduce excessive ROS production by primed microglia in brain pathology.

## Materials and Methods

### Cell Culture

All experiments were performed on BV-2 microglial cells (kindly provided by Dr. E. Blasi, Perugia, Italy), which resemble primary cultured microglia and microglia in brain tissue in their ion channel expression pattern as well as in their capability to produce ROS [10, 23, 24]. BV-2 microglial cells were cultured in FCS-containing DMEM culture medium as described previously [10]. For ROS, glutathione and nitric oxide imaging experiments, cells were plated in black 24-well plates with glass bottom (Greiner Bio One, Stonehouse, UK) at a density of 5x10^4^ cells/well. 30 min after plating, cells were treated without or with the following inhibitors as indicated: 20 μM ML133 hydrochloride (ML133; R&D systems, Abingdon, UK); 100 nM 5-iodo-resiniferatoxin (I-RTX; Alomone Lab, Jerusalem, Israel); 1 μM margatoxin (MTX; PeptaNova, Sandhausen, Germany); 20 μM 4-(4-Fluorophenyl)-2-(4-methylsulfinylphenyl)-5-(4-pyridyl)-1H-imidazole (SB203580); 20 μM 2'-Amino-3'methoxyflavone (PD98059); 5 μM N-(p-Amylcinnamoyl)anthranilic acid (ACA) (all three from Merckmillipore, Darmstadt, Germany); 1 μM 1-[(2-Chlorophenyl)diphenylmethyl]-1H-pyrazole (TRAM-34); 50 μM N-[(1R)-1,2,3,4-Tetrahydro-1-naphthalenyl]-1H-Benzimidazol-2-amine hydrochloride (NS8593); 1 mM N-Acetyl-L-cysteine (NAC) (all three from Sigma-Aldrich, Dorset, UK). 30 min after drug pretreatment, in some cases 10 ng/ml or 50 ng/ml interferon-γ (IFNγ; R&D systems, Abingdon, UK) was added to the culture medium as indicated. Thereafter, cells were incubated in a cell culture incubator at 37^°^C for 24 h, i.e., cells were primed as indicated. In our experiments, IFNγ was used at concentrations similar to those found to initiate priming of NADPH oxidase activity in human and mouse microglia [11, 12].

After priming, the culture medium was removed and cells were treated with DMEM containing the appropriate fluorescent dye with or without 2 mM Na_2_ATP (Sigma-Aldrich, Dorset, UK) at 37^°^C for 1 h to stimulate microglial ROS production. 2 mM ATP was used in these experiments, because this ATP concentration is in the physiological range [25, 26]. It has been demonstrated previously that ATP stimulates NADPH oxidase-mediated microglial ROS production via activation of P2X7 receptors [6], which have an activation threshold of 1 mM ATP. Our preliminary experiments revealed that 2 mM ATP causes significant ROS production by microglia, while this concentration is still below the ATP concentration leading to maximum ROS production (data not shown). These are optimal conditions for studying priming of microglial ROS production.

### ROS Imaging

To study microglial ROS production, cells were loaded with 10 μM CM-H_2_DCFDA (Molecular Probes, Paisley, UK) in presence or absence of ATP. In some cases, 10 μM gp91ds-tat (AnaSpec, Fremont, USA) or 200 μM Nω-Nitro-L-arginine methyl ester hydrochloride (L-NAME; Sigma-Aldrich, Dorset, UK) were added to the medium as indicated. These inhibitors were additionally present in the IFNγ priming medium for a certain time (L-NAME for 30 min; gp91ds-tat for 2 h) prior to medium exchange. Thereafter, cells were washed with extracellular medium E_1_ containing: 130 mM NaCl, 5 mM KCl, 2 mM CaCl_2_, 1 mM MgCl_2_, 100 μM GdCl_3_, 10 mM HEPES, 10 mM D-glucose (pH = 7.4). Fluorescence intensity of cells was captured using an imaging system consisting of an inverted microscope IX50 equipped with a 40X water immersion objective UApo/340 (Olympus, Hamburg, Germany), a Hamamatsu Orca 03G camera, a monochromator Polychrome V (both from Till Photonics, Munich, Germany), a dichroic mirror of 505 nm wavelength, and a barrier filter of 530±20 nm wavelength (both from Olympus, Hamburg, Germany). Cells were excited at 480 nm and images were captured for at least four different visual fields from at least two independent experiments for each condition using the Live Acquisition software (Till Photonics, Munich, Germany). Background corrected fluorescence intensities of individual cells were determined using ImageJ v1.50 (NIH, Bethesda, USA), while “n” represents the number of cells analyzed.

### Nitric Oxide Imaging

After priming, cells were treated with DMEM containing 10 μM 4-Amino-5-Methylamino-2',7'-Difluorofluorescein Diacetate (DAF-FM; ThermoFisherScientific, Paisley, UK) with or without ATP as indicated. In some cases, 200 μM L-NAME was added to the stimulation medium and was additionally present in the IFNγ priming medium for 30 min prior to medium exchange. Thereafter, cells were washed with medium E_1_. Fluorescence intensity of cells was captured using an imaging system and filter sets as described above for DCFDA-based ROS imaging. Images were captured for at least four different visual fields from at least two independent experiments for each condition using the Live Acquisition software (Till Photonics, Munich, Germany). Background corrected fluorescence intensities of individual cells were determined using ImageJ v1.50 (NIH, Bethesda, USA), while “n” represents the number of cells analyzed.

### Glutathione Imaging

To determine glutathione levels, cells were primed as indicated. Thereafter, the medium was removed and cells were exposed to DMEM containing 30 μM monochlorobimane (mBCl; Molecular Probes, Paisley, UK) for 30 min in the cell culture incubator. Following this incubation, the medium was removed and replaced with medium E_1_. Fluorescence intensity of mBCl-loaded cells was captured using the same imaging system described above, but using a dicroic mirror of 400 nm and a 420 nm long pass emission filter. Cells were excited at 380 nm using the monochromator. Images were captured for at least four different visual fields from at least two independent experiments for each condition using the Live Acquisition software (Till Photonics, Munich, Germany). Background corrected fluorescence intensities of individual cells were determined using ImageJ v1.50 (NIH, Bethesda, USA), while “n” represents the number of cells analyzed. To check the specificity of mBCL for glutathione over other thiols, 500 μM ethacrynic acid (Santa Cruz Biotechnology, Dallas, USA), an inhibitor of glutathione S-transferase, was added 10 min prior to exposure of cells to mBCl in a paired well, and mBCl fluorescence intensities were corrected for these values.

### Cell Viability

In all imaging experiments, data were collected exclusively from intact cells. Cell viability was assessed via dye loading properties of the cells. Damaged cells were defined as cells, which had lost fluorescent dyes due to loss of membrane integrity. In all experimental conditions, damaged cells were rarely found (<1% of all cells) and were excluded from further analyses.

### Quantitative RT-PCR

For quantitative RT-PCR experiments, cells were plated in 6-well plates at a density of 0.5x10^6^ cells/well and were either kept untreated or treated with 50 ng/ml IFNγ in presence or absence of inhibitors for 24 h prior to RNA isolation. Quantitative RT-PCR experiments were performed in three different cell cultures as described previously [27]. In brief, RNA was purified with E.Z.N.A Total RNA kit (Omega Bio-tek, Norcross, USA). RNA was DNAse I treated in-column during the purification process and 500 ng RNA were reverse transcribed using random hexamers and Maxima reverse transcriptase according to manufacturer’s instructions (Fisher Scientific, Hampton, USA). Quantitative PCR was conducted on a C1000 Thermal Cycler (Biorad, Hercules, USA) with 30 ng of reverse transcribed RNA and DyNAmo Flash SYBR Green qPCR mix (Thermo Scientific, Walham, USA) using the following mouse specific primers: NOX1 (Forward, 5′-AATGCCCAGGATCGAGGT-3′; Reverse, 5′-GATGGAAGCAAAGGGAGTGA-3′), NOX2 (Forward, 5′-CCCTTTGGTACAGCCAGTGAAGAT-3′; Reverse, 5′-CAATCCCGGCTCCCACTAACATCA-3′), NOX4 (Forward, 5′-GGATCACAGAAGGTCCCTAGCAG-3′; Reverse, 5′-GCGGCTACATGCACACCTGAGAA-3′), L7 (Forward, 5’-GAAGCTCATCTATGAGAAGGC-3’; Reverse, 5’-AAGACGAAGGAGCTGCAGAAC-3’). Primers for the ribosomal protein L7 RNA were used as a control.

### Statistics

All data are presented as mean ± SEM. Statistical significance of differences between experimental groups was evaluated using SPSS v22 (IBM, Armonk, USA). For data analysis of qPCR experiments, Student’s t-test was used. In all other cases, one-way ANOVA with Dunnett’s T3 post hoc tests was used after testing for equal variances with Levene’s test. Data were considered to be statistically significant with p<0.05.

## Results

### IFNγ-Induced Priming of Microglial ROS Production

**Fig 1:**
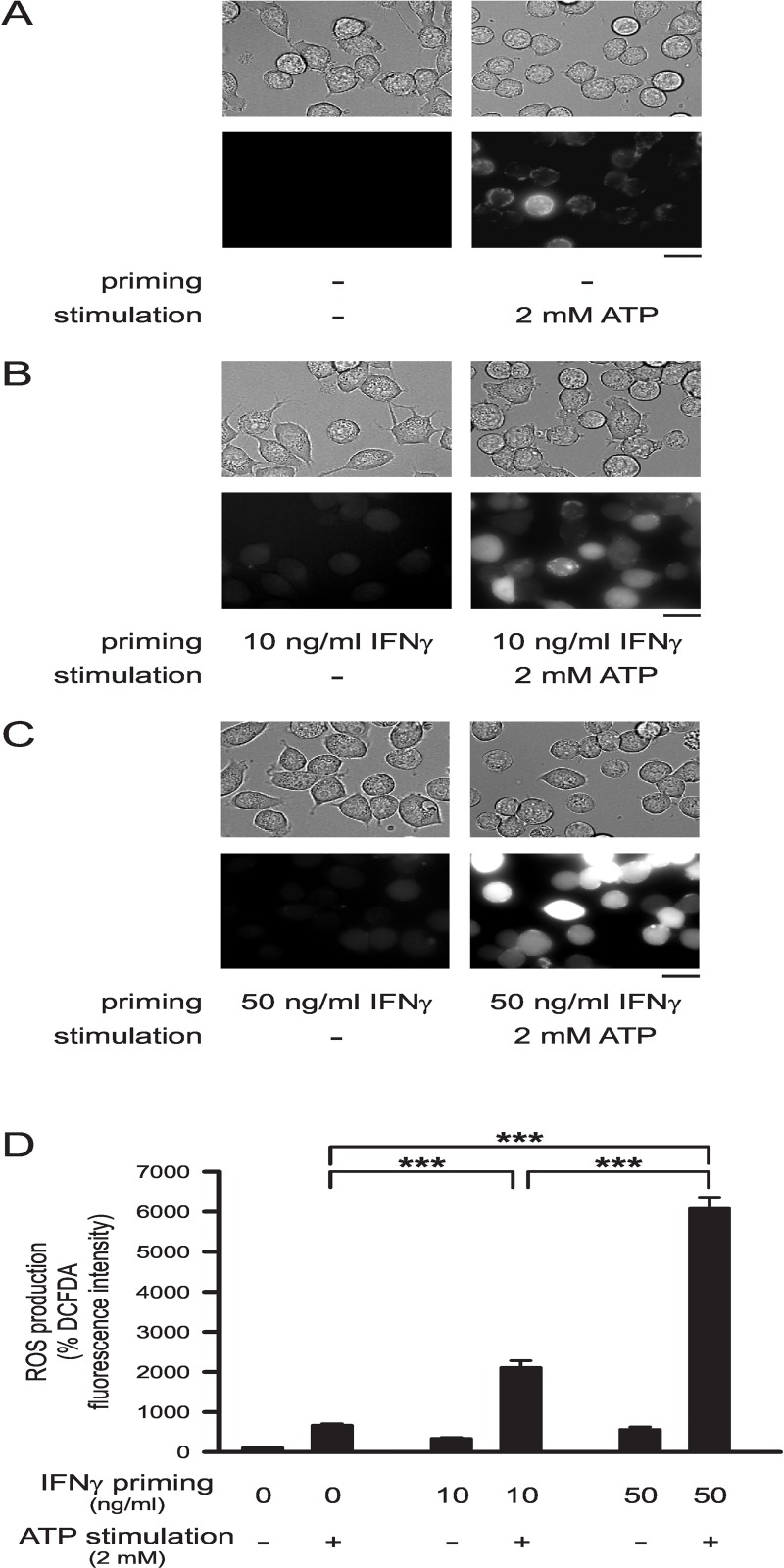
IFNγ-induced priming of ATP-stimulated ROS production by microglia. (A-C) Examples of cells (upper images: brightfield images; lower images: DCFDA fluorescence images) kept unprimed (A) or primed with 10 ng/ml IFNγ (B) or 50 ng/ml IFNγ (C) for 24 h and maintained subsequently with or without 2 mM ATP for 1 h. Scale bars: 20 γm. (D) Mean DCFDA fluorescence intensities of cells maintained unprimed for 24 h and then either kept unstimulated (n = 1134) or stimulated with 2 mM ATP (n = 1137) for 1 h, of cells primed for 24 h with 10 ng/ml IFNγ and then not stimulated (n = 216) or stimulated with 2 mM ATP (n = 264) for 1 h, or cells primed for 24 h with 50 ng/ml IFNγ and subsequently kept unstimulated (n = 240) or stimulated with 2 mM ATP (n = 1089) for 1 h. Mean DCFDA fluorescence intensities of cells were normalized to the mean fluorescence intensity determined for unprimed and unstimulated control cells. ***, p<0.001.

Fig 1 demonstrates brightfield images and DCFDA fluorescence images of microglial cells, which were kept untreated or were treated with 2 mM ATP for 1 h following pretreatment with IFNγ for 24 h. Analysis of DCFDA fluorescence intensities as a measure for microglial ROS production revealed significant increases in microglial cells stimulated with ATP. Without IFNγ priming, ROS production of ATP-stimulated cells was 6.5-fold higher than that of unstimulated cells (p<0.001; Fig 1A, Fig 1D). Priming of microglial cells with IFNγ significantly increased DCFDA staining intensities of cells, i.e., further enhanced microglial ROS production following ATP stimulation. In comparison with cells kept unprimed, ATP-stimulated ROS production was increased 3.2-fold (p<0.001; Fig 1B, Fig 1D) and 9.3-fold (p<0.001; Fig 1C, Fig 1D) in cells primed with 10 ng/ml IFNγ or 50 ng/ml IFNγ, respectively. In the absence of ATP stimulation, IFNγ pretreatment did not induce substantial ROS production (Fig 1).

**Fig 2:**
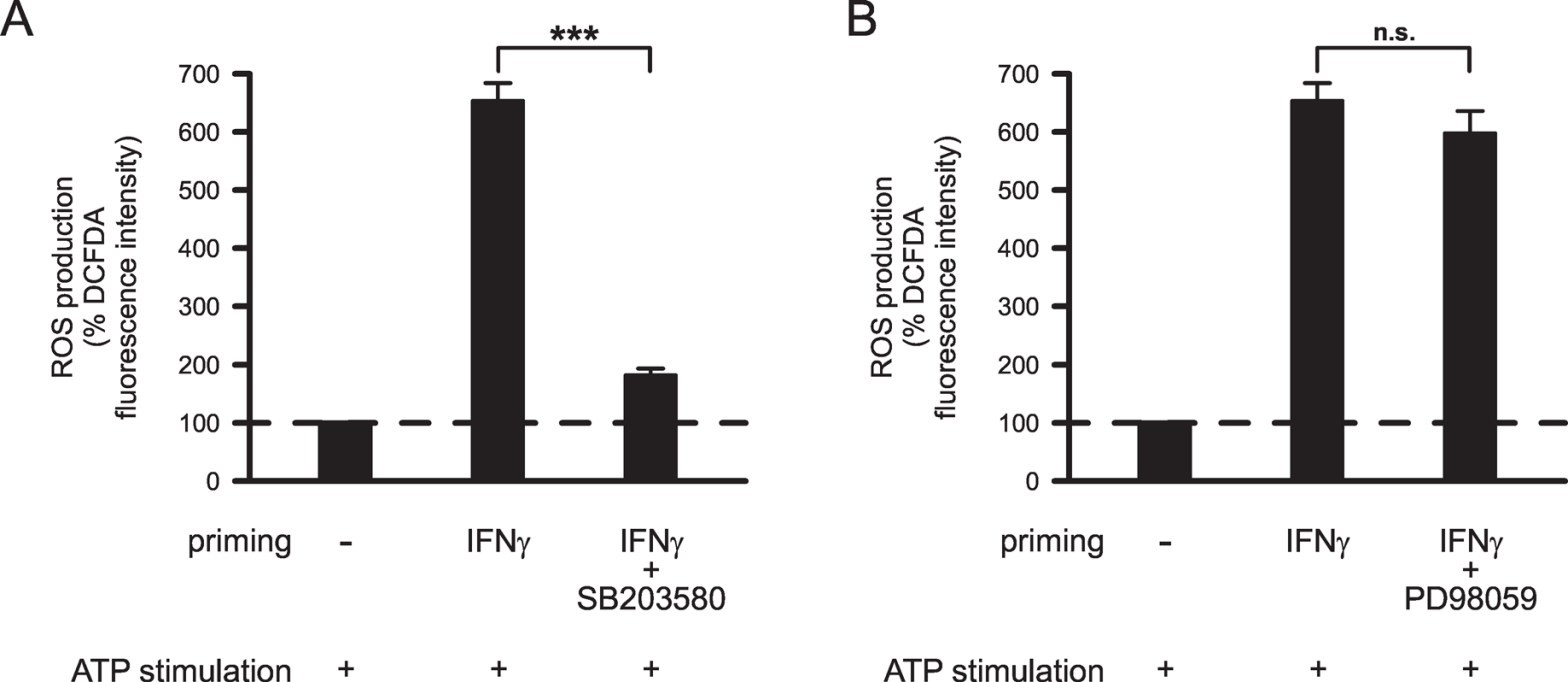
Effects of inhibitors of p38 MAPK and p42/44 ERK1/2 kinase activity on IFNγ-induced priming of ATP-stimulated ROS production by microglia. Cells were primed for 24 h with 50 ng/ml IFNγ in absence or presence of p38 MAPK inhibitor 20 μM SB203580 (A; n = 323 no priming, n = 247 IFNγ, n = 213 IFNγ+SB203580), or p42/44 ERK1/2 kinase inhibitor 20 μM PD98059 (B; n = 323 no priming, n = 247 IFNγ, n = 228 IFNγ+PD98059). After individual priming, all cells were stimulated with 2 mM ATP for 1 h. Mean DCFDA fluorescence intensities of cells were normalized to the mean fluorescence intensities determined for unprimed ATP-stimulated cells. ***, p<0.001; n.s., not significant.

Additional experiments were performed in order to determine mechanisms underlying IFNγ-induced priming of microglial ROS production. Microglia were primed with 50 ng/ml IFNγ to achieve a good signal-to-noise ratio in these fluorescence imaging experiments. To identify intracellular signaling pathways involved in IFNγ-induced priming of microglial ROS production, effects of the p38 MAPK inhibitor SB203580 and of the p42/44 ERK1/2 inhibitor PD98059 were investigated. Fig 2 shows ATP-stimulated ROS production in cells primed with IFNγ in presence or absence of either 20 μM SB203580 or 20 μM PD98059. Inhibition of p38 MAPK activity almost completely inhibited the IFNγ-induced priming effect (p<0.001; Fig 2A), whereas inhibition of ERK1/2 activity did not significantly affect IFNγ-induced priming of microglial ROS production (p = 1.0; Fig 2B).

**Fig 3:**
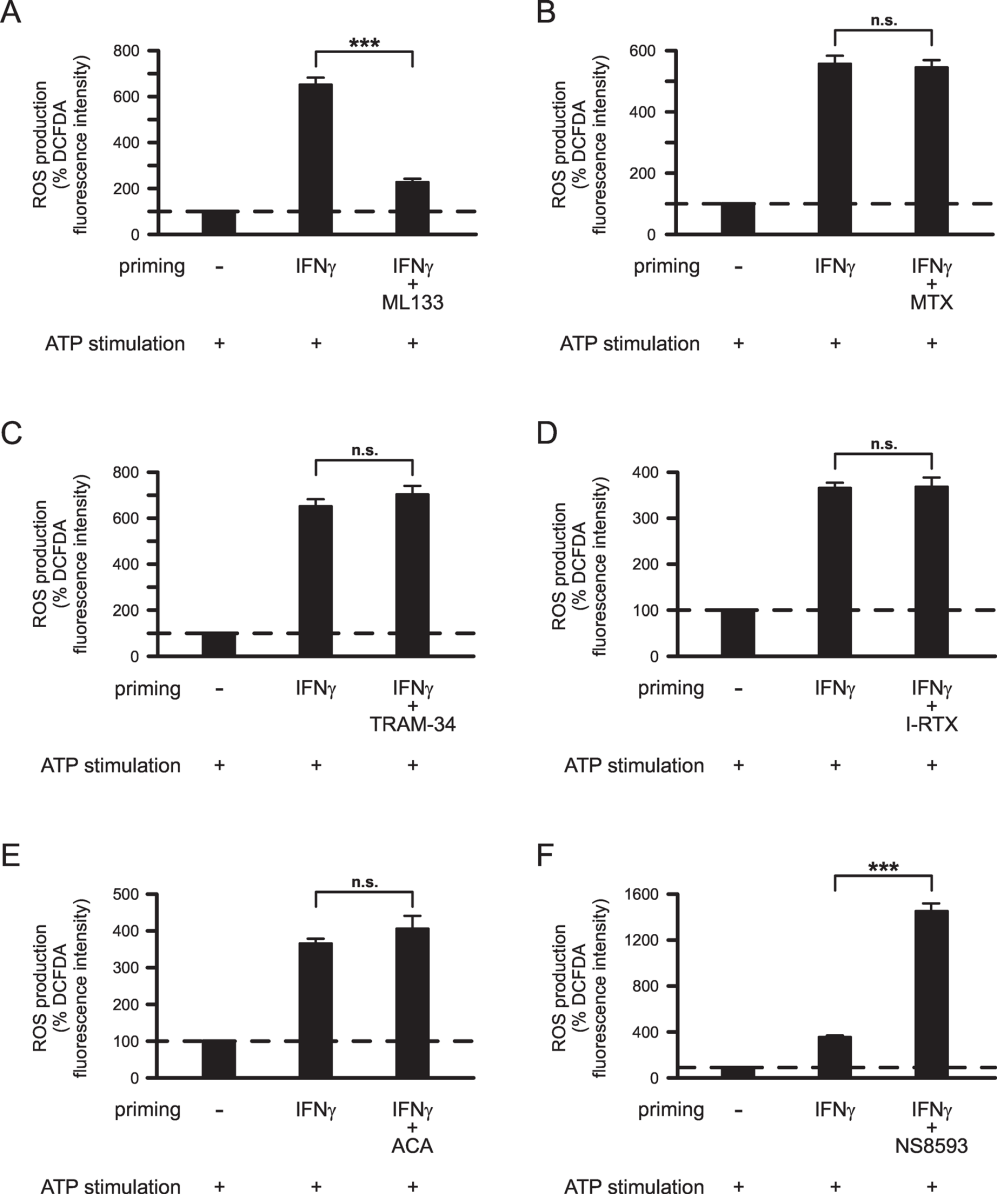
Effects of K^+^ and TRP channel inhibitors on IFNγ-induced priming of ATP-stimulated ROS production by microglia. Prior to stimulation, microglia were primed for 24 h without or with 50 ng/ml IFNγ and K^+^ channel inhibitors 20 μM ML133 (A; n = 323 no priming, n = 247 IFNγ, n = 259 IFNγ+ML133), 1 μM MTX (B; n = 199 no priming, n = 191 IFNγ, n = 223 IFNγ+MTX) and 1 μM TRAM-34 (C; n = 323 no priming, n = 247 IFNγ, n = 295 IFNγ+TRAM-34) or TRP channel inhibitors 100 nM I-RTX (D; n = 282 no priming, n = 280 IFNγ, n = 300 IFNγ+I-RTX), 5 μM ACA (E; n = 282 no priming, n = 280 IFNγ, n = 190 IFNγ+ACA) and 50 μM NS8593 (F; n = 282 no priming, n = 280 IFNγ, n = 183 IFNγ+NS8593). Following priming, all cells were stimulated with 2 mM ATP for 1 h. Mean DCFDA fluorescence intensities of cells were normalized to mean fluorescence intensities determined for unprimed ATP-stimulated cells. ***, p<0.001; n.s., not significantly different.

Next, we investigated the role of K^+^ and TRP channels in priming of microglial ROS production. In these experiments, microglial cells were primed with 50 ng/ml IFNγ in presence or absence of ion channel inhibitors. Blockade of Kir2.1 inward rectifier K^+^ channels with 20 μM ML133 inhibited IFNγ-induced priming of ATP-stimulated ROS production by 76.8% (p<0.001; Fig 3A). In contrast, blockade of Kv1.3 voltage-gated outward rectifier K^+^ channels with 1 μM MTX (p = 1.0; Fig 3B) or inhibition of KCa3.1 IK-type Ca^2+^-activated K^+^ channels with 1 μM TRAM-34 did not significantly alter the IFNγ-induced priming effect (p = 1.0; Fig 3C). Furthermore, none of the tested TRP channel inhibitors attenuated the priming effect of IFNγ on microglial ROS production. No significant differences in ROS production were observed between ATP-stimulated cells primed with 50 ng/ml IFNγ in absence or presence of TRPV1 channel inhibitor I-RTX (100 nM; p = 1.0; Fig 3D) or TRPM2 channel inhibitor ACA (5 μM; p = 0.998; Fig 3E). Intriguingly, inhibition of TRPM7 channels with 50 μM NS8593 further enhanced the priming effect of IFNγ on microglial ROS production. In comparison with cells primed with IFNγ alone, ROS production by ATP-stimulated microglial cells was increased 4-fold (p<0.001) in cells primed with IFNγ in the presence of NS8593 (Fig 3F).

Control experiments revealed that SB203580 and ML133 selectively inhibited priming of microglial ROS production without having direct inhibitory effects on ATP-stimulated ROS production. 24 h-lasting pretreatment of cells with SB203580 or ML133 in the absence of IFNγ did not inhibit microglial ATP-stimulated ROS production (p = 0.565, n = 214 for SB203580; p = 1.0, n = 211 for ML133; data not shown).

In summary, these data suggest that IFNγ induces priming of microglial ATP-stimulated ROS production, which involves activity of p38 MAPK and Kir2.1 inward rectifier K^+^ channels. Further experiments aimed (i) to identify mechanisms causing priming of microglial ROS production and (ii) to elucidate mechanisms by which inhibitors of p38 MAPK and Kir2.1 K^+^ channels modulate the IFNγ-induced priming effect.

### IFNγ-Induced Changes in Microglial Glutathione Levels

**Fig 4:**
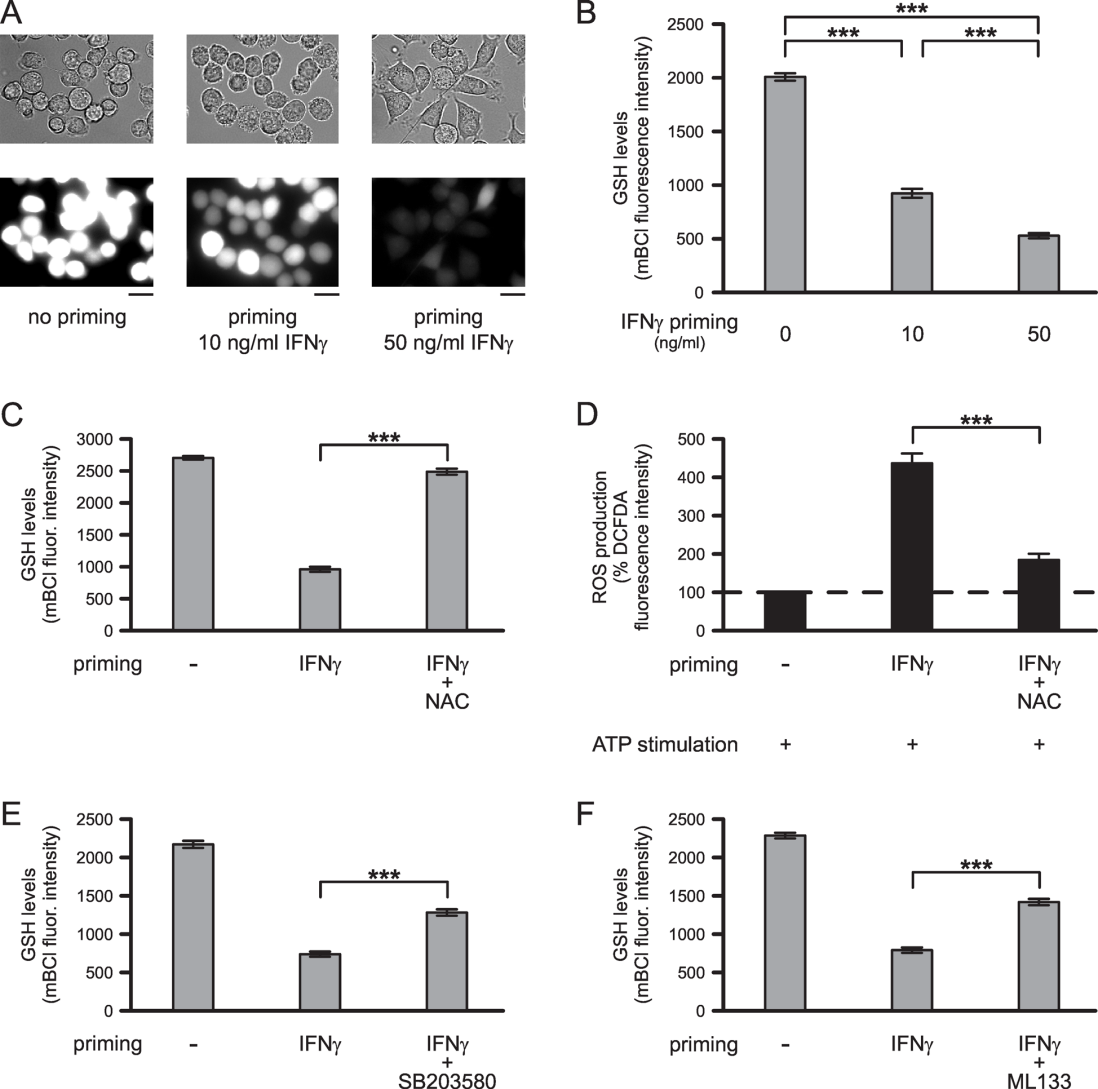
IFNγ-induced changes in microglial GSH levels. (A) Examples of cells (upper lane: brightfield images; lower lane: mBCl fluorescence images) kept unprimed or primed with 10 ng/ml IFNγ or 50 ng/ml IFNγ for 24 h. Scale bars: 20 μm. (B) Mean mBCl fluorescence intensities of cells maintained unprimed (n = 423) or primed with 10 ng/ml IFNγ (n = 295) or 50 ng/ml IFNγ (n = 370) for 24 h. (C) Intracellular GSH levels, i.e., mean mBCl fluorescence intensities of cells maintained unprimed or primed for 24 h with 50 ng/ml IFNγ in absence or presence of 1 mM NAC (n = 389 no priming, n = 421 IFNγ, n = 242 IFNγ+NAC). (D) Effects of 1 mM NAC on IFNγ-induced priming of microglial ROS production. Cells were kept unprimed or were primed for 24 h with IFNγ in absence or presence of 1 mM NAC as indicated (n = 298 no priming, n = 291 IFNγ, n = 277 IFNγ+NAC). Following priming, all cells were stimulated with 2 mM ATP for 1 h. Mean DCFDA fluorescence intensities of cells were normalized to mean fluorescence intensities determined for unprimed ATP-stimulated cells. (E-F) Effects of kinase and K^+^ channel inhibitors on IFNγ-induced changes in microglial GSH levels. Cells were primed for 24 h without or with 50 ng/ml IFNγ and 20 μM SB203580 (E; n = 272 no priming, n = 279 IFNγ, n = 252 IFNγ+SB203580) or 20 μM ML133 (F; n = 289 no priming, n = 258 IFNγ, n = 259 IFNγ+ML133). ***, p<0.001.

IFNγ priming of microglia causes a reduction in the levels of intracellular glutathione (GSH), which is one of the most abundant antioxidants in microglia [4]. Fig 4A demonstrates mBCl fluorescence images as a measure of GSH levels in untreated microglia and in microglia treated for 24 h with either 10 ng/ml IFNγ or 50 ng/ml IFNγ. Quantitative analyses of mBCl fluorescence intensities revealed that IFNγ reduced GSH levels in microglia in a concentration-dependent manner. In comparison with untreated microglia, GSH levels were reduced by 53.9% (p<0.001) or by 73.3% (p<0.001) following 24 h-lasting exposure of cells to 10 ng/ml IFNγ or 50 ng/ml IFNγ, respectively (Fig 4B).

Next, we aimed to clarify whether augmented ROS levels determined in ATP-stimulated microglia following IFNγ priming were due to reduced GSH levels, i.e., due to reduced antioxidant capability of the cells. Therefore, we increased intracellular glutathione levels and tested whether this procedure affects IFNγ-induced priming of microglial ROS production. As shown in Fig 4C, addition of NAC to IFNγ-containing priming medium almost completely reversed effects of IFNγ on intracellular GSH levels. In cells primed simultaneously with IFNγ and 1 mM NAC for 24 h, GSH levels were increased by 87.3% (p<0.001) compared to those determined in IFNγ-primed microglia. Fig 4D demonstrates that elevation of intracellular GSH levels prevented the IFNγ-mediated priming effect on ATP-stimulated ROS production. In comparison with cells primed exclusively with IFNγ, ATP-stimulated ROS production was reduced by 74.7% (p<0.001) in cells primed with IFNγ in the presence of 1 mM NAC.

Furthermore, we tested whether inhibitory effects of SB203580 and/or ML133 on priming of microglial ROS production were due to modulation of intracellular GSH levels. As shown in Fig 4, inhibition of p38 MAPK with SB203580 (Fig 4E) or blockade of Kir2.1 K^+^ channels with ML133 (Fig 4F) partially reversed the IFNγ-induced reduction of microglial GSH levels. In comparison with microglia primed with IFNγ alone, GSH levels of microglia primed with IFNγ and SB203580 were 37.5% higher (p<0.001), while GSH levels of microglia primed with IFNγ and ML133 were 42.2% higher (p<0.001) than those determined for cells primed with IFNγ in the absence of either SB203580 or ML133. In the absence of IFNγ, GSH levels of microglia treated with SB203580 or ML133 for 24 h were not significantly affected (p = 0.283, n = 223 for SB203580; p = 0.955, n = 189 for ML133; data not shown).

### IFNγ-Induced Changes in Microglial NOX Subunit Expression

**Fig 5:**
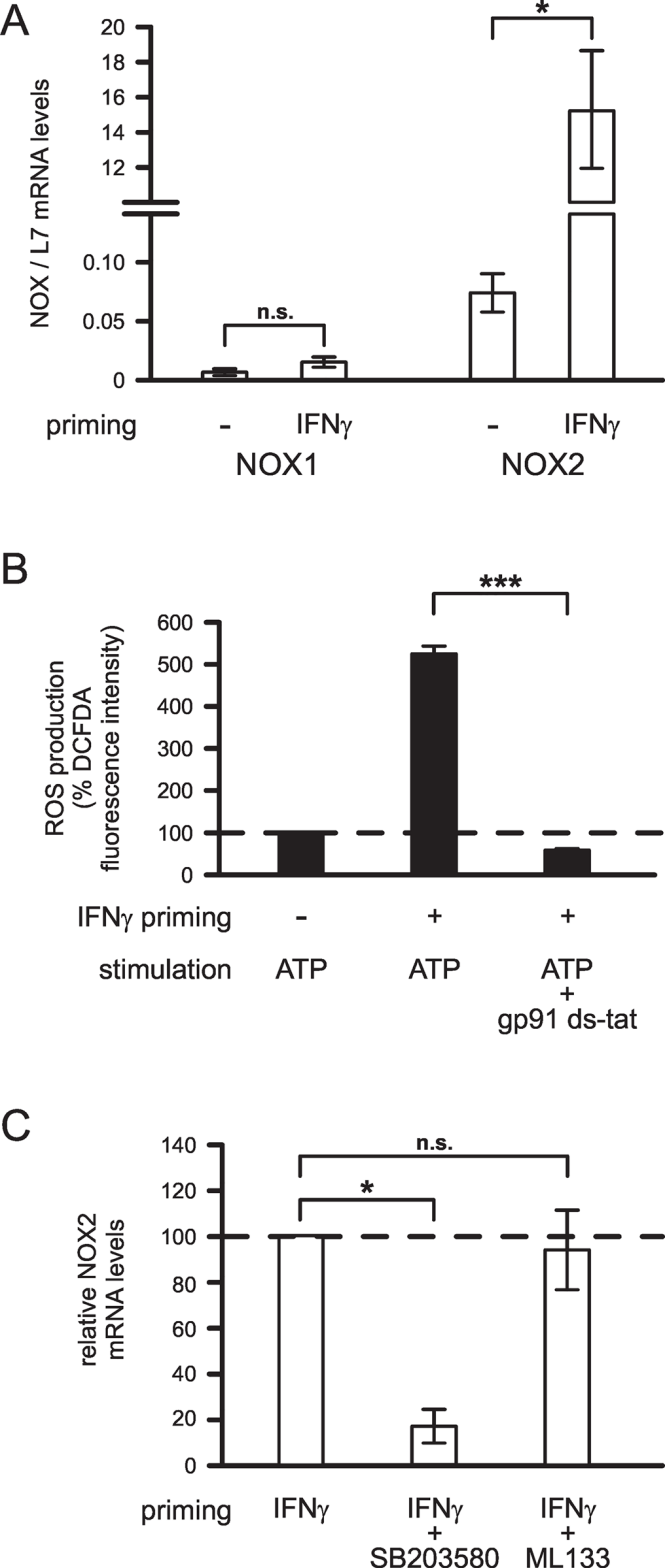
IFNγ-induced changes in microglial expression levels of NOX subunits. (A) Expression of NOX1 and NOX2 in cells kept untreated or treated with 50 ng/ml IFNγ for 24 h. Using qPCR, NOX1 and NOX2 mRNA levels were determined and normalized to L7 mRNA levels. (B) Effects of NOX2 inhibitor gp91ds-tat on microglial ATP-stimulated ROS production (n = 455 no priming, ATP stimulation; n = 338 IFNγ priming, ATP stimulation; n = 363 IFNγ priming, ATP+gp91ds-tat stimulation). Prior to stimulation, microglia were primed for 24 h without or with 50 ng/ml IFNγ. Following priming, cells were stimulated with 2 mM ATP in absence or presence of 10 μM gp91ds-tat for 1 h. Mean DCFDA fluorescence intensities of cells were normalized to mean fluorescence intensities determined for unprimed ATP-stimulated cells. (C) Relative NOX2 mRNA levels determined in microglia treated with 50 ng/ml IFN-γ for 24 h in absence or presence of 20 μM SB203580 or 20 μM ML133 as indicated. ***, p<0.001; *, p<0.05; n.s., not significantly different.

We next investigated which NADPH oxidase subunits were expressed and upregulated by IFNγ in microglia, and tested whether IFNγ-induced changes in expression levels of NADPH oxidase subunit(s) were affected by SB203580 and/or ML133. To date, three NOX subunits have been identified in microglia, namely NOX1, NOX2 and NOX4 [2]. To determine IFNγ-induced changes in microglial NOX1, NOX2 and NOX4 mRNA levels, we used RT-qPCR. In comparison with cells kept untreated, microglia primed with 50 ng/ml IFNγ for 24 h resulted in a significant increase in NOX2 mRNA levels (205-fold; p<0.05; Fig 5A). In contrast to the expression of NOX2 mRNA, expression of NOX1 mRNA was very low and did not change significantly (p = 0.170) following priming of cells with IFNγ (Fig 5A). In all three independent experiments, expression of NOX4 mRNA was undetectable at each of the experimental conditions tested.

To verify the priming effect of IFNγ on NOX2 expression, we additionally tested effects of the specific NOX2 inhibitor gp91ds-tat (10 μM) on ATP-stimulated ROS production in IFNγ-primed microglia. As demonstrated in Fig 5B, IFNγ-induced upregulation of ATP-stimulated ROS production was abolished by gp91ds-tat (p<0.001), suggesting that enhanced ROS production of IFNγ-primed microglia was mediated exclusively by the activity of NADPH oxidase subunit NOX2.

To identify the role of p38 MAPK and Kir2.1 K^+^ channels in regulating NOX2 levels in IFNγ-primed microglia, we additionally investigated effects of SB203580 and ML133 on microglial NOX2 mRNA expression. As demonstrated in Fig 5C, NOX2 mRNA levels of IFNγ-primed microglia were reduced by 82.7% (n = 3; p<0.05) upon inhibition of p38 MAPK with SB203580. In contrast, K^+^ channel inhibition with ML133 (n = 3; p = 1.0) did not affect NOX2 mRNA levels in IFNγ-primed microglia (Fig 5C).

### IFNγ-Induced Changes in Microglial NO Production

**Fig 6:**
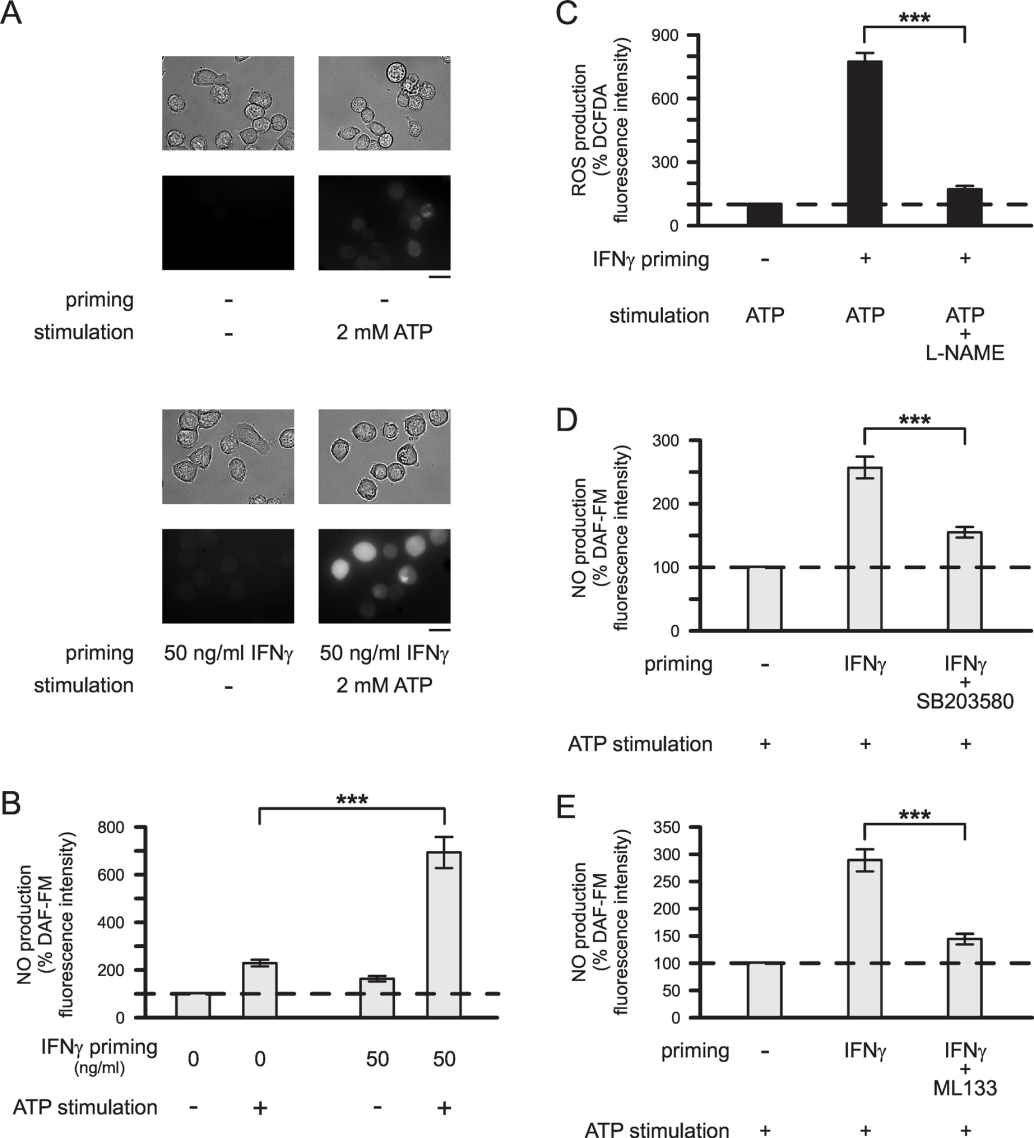
IFNγ-induced changes in microglial NO production. (A) Examples of cells (upper images: brightfield images; lower images: DAF-FM fluorescence images) kept unprimed or primed with 50 ng/ml IFNγ for 24 h and maintained subsequently with or without 2 mM ATP for 1 h. Scale bars: 20 μm. (B) Mean DAF-FM fluorescence intensities of cells maintained unprimed for 24 h and then either kept unstimulated (n = 288) or stimulated with 2 mM ATP (n = 240) for 1 h, or of cells primed for 24 h with 50 ng/ml IFNγ and subsequently kept unstimulated (n = 304) or stimulated with 2 mM ATP (n = 224) for 1 h. (C) Inhibitory effects of 200 μM L-NAME on ATP-stimulated ROS production by IFNγ-primed microglia (n = 371 no priming, ATP stimulation; n = 268 IFNγ priming, ATP stimulation; n = 236 IFNγ priming, ATP+L-NAME stimulation). (D-E) Inhibitory effects of 20 μM SB203580 (D; n = 156 no priming; n = 153 IFNγ priming; n = 185 IFNγ+SB203580 priming) and 20 μM ML133 (E; n = 240 no priming; n = 224 IFNγ priming; n = 262 IFNγ+ML133 priming) on IFNγ-induced enhancement of microglial ATP-stimulated NO production. (C-E) Prior to stimulation, microglia were primed for 24 h without or with 50 ng/ml IFNγ in absence or presence of inhibitors as indicated. Following priming, cells were stimulated for 1 h with 2 mM ATP in absence or presence of inhibitors. Mean fluorescence intensities of cells were normalized to mean fluorescence intensities determined for unprimed ATP-stimulated cells. ***, p<0.001.

As demonstrated in Fig 6A and Fig 6B, ATP-stimulated NO production of microglial cells was also enhanced following IFNγ priming. Stimulation of unprimed microglia with 2 mM ATP for 1 h increased DAF-FM fluorescence intensities as a measure for NO production 2.3-fold (p<0.001) compared to unstimulated cells. In microglia primed with 50 ng/ml IFNγ for 24 h, ATP-stimulated NO production was 2.9-fold (p<0.001) higher than in unprimed ATP-stimulated cells (Fig 6B). As demonstrated in Fig 6C, inhibition of NO production almost completely inhibited ATP-stimulated ROS production of IFNγ-primed microglia. It was reduced by 89.2% (p<0.001) in the presence of 200 μM L-NAME. These data suggest that ATP stimulation of IFNγ-primed microglial cells mainly leads to the formation of peroxynitrite, a ROS detectable by the fluorescent dye DCFDA.

NO production of IFNγ-primed microglia was significantly inhibited upon p38MAPK inhibition or Kir2.1 K^+^ channel blockade. In cells primed with IFNγ and SB203580, ATP-stimulated NO production was reduced by 41.7% (p<0.001) in comparison with cells primed with IFNγ in the absence of SB203580 (Fig 6D). Furthermore, in comparison with cells primed with IFNγ alone, ATP-stimulated NO production was significantly reduced by 51.4% (p<0.001) in microglial cells primed with IFNγ in the presence of ML133 (Fig 6E). Control experiments revealed that SB203580 and ML133 did not have a direct inhibitory effect on microglial NO production. Statistically significant differences were not found in ATP-stimulated NO production between unprimed cells and cells primed for 24 h with SB203580 or ML133 in the absence of IFNγ (p = 0.352, n = 225 for SB203580; p = 0.421, n = 172 for ML133; data not shown).

## Discussion

In this study, we elucidated mechanisms underlying priming of microglial ROS production. We demonstrate that IFNγ-induced priming simultaneously stimulates three mechanisms, namely (i) upregulation of NADPH oxidase subunit NOX2, (ii) upregulation of NO production and (iii) reduction of intracellular GSH levels. All three mechanisms were found to be dependent on p38 MAPK activity, whereas p42/p44 ERK was not involved. Furthermore, by testing a variety of ion channel inhibitors, we identified Kir2.1 inward rectifier K^+^ channels as crucial regulators of IFNγ-induced priming of microglial ROS production. Blockade of Kir2.1 channels inhibited effects of IFNγ on GSH levels and NO production. However, unlike p38 MAPK inhibition, Kir2.1 channel inhibition did not affect the upregulation of NOX2. These data suggest that IFNγ-induced p38 MAPK and Kir2.1 inward rectifier K^+^ channel activity are not directly linked.

In agreement with previous studies, treatment with ATP immediately stimulated microglial ROS production [6], whereas the priming agent IFNγ did not induce substantial ROS or NO production [11, 12]. Furthermore, our data demonstrate that IFNγ priming potentiates the production of ROS and NO by ATP-stimulated microglia. In previous studies addressing IFNγ-induced priming of ROS production, microglial cells were stimulated with the phorbol ester PMA [11, 12], which exclusively activates the NADPH oxidase. To mimic pathophysiological situations in vivo, we used ATP to induce microglial ROS production. For example, traumatic brain injury and stroke are accompanied by substantial damage of neurons, which contain ATP at millimolar concentrations [25, 26]. Our data suggest that ATP stimulation of IFNγ-primed microglia activate both NADPH oxidase NOX2 and inducible nitric oxide synthase (iNOS) causing simultaneous generation of superoxide anion and NO, which subsequently leads to the formation of peroxynitrite. Previous studies have demonstrated that peroxynitrite is more neurotoxic than superoxide or NO alone [4]. Reduction of glutathione levels following priming of microglia with IFNγ further leads to increased peroxynitrite concentrations, while addition of NAC to the external medium attenuated both IFNγ-induced reduction in intracellular GSH levels and IFNγ-induced enhancement of ATP-stimulated ROS production. Reduced glutathione levels have been found in primed microglia isolated from the aged brain [28]. Furthermore, in brains of patients with neurodegenerative diseases, including Alzheimer’s disease and Parkinson’s disease, increased peroxynitrite production and reduced GSH levels have been found [4, 29]. Thus, pharmacological tools inhibiting both enhanced peroxynitrite formation and reduction in GSH levels would provide a promising strategy to reduce microglia-mediated oxidative stress in the brain of aged people and patients with neurodegenerative diseases.

Microglial ion channels have recently been identified as potential therapeutic targets in neurological diseases [24, 30, 31], while K^+^, H^+^, transient receptor potential (TRP) and Cl^-^ channels have been found to regulate microglial production of ROS [10, 32–37]. In this study, we elucidated a novel role of microglial Kir2.1 inward rectifier K^+^ channels, namely regulation of microglial priming processes, which lead to enhanced microglial ROS production. We demonstrate for the first time that Kir2.1 inward rectifier K^+^ channels are crucially involved in mechanisms leading to reduced intracellular glutathione levels and upregulated NO production, two processes contributing to IFNγ-induced priming of ROS production by ATP-stimulated microglia. Kir2.1 inward rectifier K^+^ channels are expressed in rodent [38] and human microglia [39]. Intriguingly, inward rectifier K^+^ channel expression is upregulated in microglia of aged mice [40].

Kir2.1 inward rectifier K^+^ channels mainly regulate the resting membrane potential of microglial cells, i.e., channel activity causes membrane hyperpolarization [24]. It is possible that a negative membrane potential is a prerequisite to initiating or to maintaining microglial priming processes. In microglia, IFNγcauses Ca^2+^ influx from the extracellular medium [41]. Increases in intracellular Ca^2+^ concentration stimulate activity of Ca^2+^/calmodulin-dependent protein kinase II, which is involved in IFNγ-induced JAK/STAT1 pathway leading to increased iNOS expression and subsequent NO production [42, 43]. Inhibition of microglial Kir2.1 channels causes a depolarization-mediated decrease in the driving force for Ca^2+^ influx [44], which could be responsible for the observed attenuation of NO production. The precise mechanisms by which Kir2.1 channel activity regulates NO production and intracellular glutathione levels remain to be elucidated. It can be excluded that Kir2.1 channels regulate the activity of p38 MAPK, as NOX2 upregulation could only be inhibited by SB203580, but not by ML133.

In microglia, voltage-gated outward rectifier Kv1.3 channels have recently been found to regulate NADPH oxidase priming induced by soluble amyloid-β [10]. In contrast, IFNγ-induced priming of microglial ROS production does not require activity of Kv1.3 channels. Even at a concentration 10-times higher than that used in the previous study by Schilling and Eder [10], MTX did not affect IFNγ-induced priming of ATP-stimulated ROS production by microglial cells (present study). These data suggest that K^+^ channel activity is required for optimal priming of microglial ROS production, while the involvement of a specific K^+^ channel type in microglial priming is stimulus-dependent.

In brain pathology, oxidative stress causing neuronal damage is mainly due to NADPH oxidase-mediated excessive ROS production by activated microglia [1, 2]. Therefore, targeting microglial NADPH oxidase NOX2 has been suggested as a promising strategy to prevent or reduce oxidative stress-induced damage of brain tissue in neurological diseases, which are accompanied by neuroinflammation [2, 45, 46]. However, complete inhibition of microglial ROS production may not only affect neurotoxic effects, but also beneficial/neuroprotective effects, of microglial cells. Blocking NOX2 could reduce antimicrobial effects leading to an increased risk of infections and/or could inhibit important signaling pathways, which are regulated by ROS and may contribute to microglial neuroprotective actions. In contrast, targeting exclusively microglial priming processes would not abolish microglial ROS production, but regulate the amount of ROS produced by activated microglia. It may provide a novel strategy to selectively reduce microglia-mediated enhanced ROS production and subsequent oxidative stress in brain pathology.

In summary, microglia priming leading to excessive ROS production upon secondary stimulation represents a major risk factor for the development of neurodegenerative diseases. A better understanding of mechanisms underlying priming of microglial ROS production may lead to the development of strategies aiming at the reduction of microglia-induced neurotoxicity. We suggest that inhibition of Kir2.1 channels provides a potential therapeutic strategy to reduce microglial priming and subsequent enhanced oxidative stress in brain pathology.
